# Voice Outcomes in Patients With Laryngopharyngeal Reflux: A Systematic Review and Meta-Analysis

**DOI:** 10.7759/cureus.90357

**Published:** 2025-08-18

**Authors:** Jaisingh Rajput, Cheikh S Mballo, Mohamed Elahtem, Amal Malik, Abdul Rafay Mahmood, Salman Niaz Ahmed, Bhavna Singla, Sri Pranita Cherukuri

**Affiliations:** 1 Family Medicine, Montgomery Family Medicine Residency Program, Baptist Health, Montgomery, USA; 2 Medicine, David Geffen School of Medicine, UCLA Health, Los Angeles, USA; 3 College of Medicine, Qatar University, Doha, QAT; 4 Medicine, Health Avenue, Lahore, PAK; 5 Internal Medicine, Akhtar Saeed Medical and Dental College, Lahore, PAK; 6 Otolaryngology - Head and Neck Surgery, Dow University Hospital, Karachi, PAK; 7 Internal Medicine, Erie County Medical Center (ECMC) Hospital, Buffalo, USA; 8 Internal Medicine, Columbia University Mailman School of Public Health, New York City, USA

**Keywords:** gastroesophageal reflux, laryngopharyngeal reflux, outcome assessment, systamatic review and meta-analysis, voice disorders

## Abstract

Laryngopharyngeal reflux (LPR) commonly causes voice disturbances, yet treatment efficacy remains debated. This review synthesizes evidence on voice outcomes in patients with LPR. A Preferred Reporting Items for Systematic reviews and Meta-Analyses (PRISMA)-guided systematic review (2000-2024) identified eight studies evaluating pharmacological, behavioral, and diagnostic approaches. Random-effects meta-analyses were conducted to assess effect sizes (ES), heterogeneity (I²), and subgroup differences. Behavioral interventions (e.g., Lee Silverman Voice Treatment {LSVT}, voice therapy) demonstrated robust efficacy (ES=0.60, 95% CI: 0.45-0.76, I²=0%), whereas proton pump inhibitors (PPIs) showed negligible effects (ES = -0.15). Adjunctive rikkunshito significantly improved outcomes in refractory LPR cases (ES=0.82). Diagnostic methods based on acoustic analysis or the Voice Handicap Index (VHI) yielded more reliable outcomes (ES=0.56) than those based on clinical symptoms or the Reflux Symptom Index (RSI) (ES=0.32, I²=94.45%). Long-term benefits beyond six months were inconsistent (ES=0.25, I²=97.47%). Behavioral therapies outperform pharmacological treatments in managing LPR-related voice dysfunction, though diagnostic precision and long-term adherence remain critical. Future studies should prioritize standardized diagnostic protocols and investigate multimodal therapeutic strategies.

## Introduction and background

Laryngopharyngeal reflux (LPR) is characterized by the retrograde flow of gastric contents into the laryngopharynx, leading to inflammation and mucosal damage [1]. Unlike gastroesophageal reflux disease (GERD), which primarily causes heartburn and regurgitation, LPR presents with extra-esophageal symptoms such as chronic cough, hoarseness, globus sensation, and frequent throat clearing [2]. Voice disturbances are among the most common complaints in patients with LPR, significantly affecting their quality of life and professional functioning, particularly in voice-dependent occupations [3].

The pathophysiology of LPR-related voice dysfunction involves direct mucosal injury from pepsin and acid, resulting in edema, erythema, and vocal fold lesions [4]. Diagnosis remains challenging due to the absence of a gold-standard test, often relying on symptom-based questionnaires (e.g., the Reflux Symptom Index) and laryngoscopic findings [5]. Treatment typically includes lifestyle modifications, proton pump inhibitors (PPIs), and dietary adjustments, though patient response is highly variable [6].

Despite growing recognition of the condition, the impact of LPR on voice outcomes remains insufficiently studied, with conflicting evidence regarding treatment effectiveness [7]. Some studies report significant improvements in vocal parameters following treatment, while others show minimal or no change [8]. This systematic review aimed to synthesize existing evidence on voice outcomes in LPR patients, with a focus on diagnostic approaches, therapeutic interventions, and long-term prognosis.

## Review

Methodology

The Preferred Reporting Items for Systematic reviews and Meta-Analyses (PRISMA) guidelines were used to conduct this systematic review, aiming to comprehensively identify relevant studies published between 2000 and 2024. Studies were eligible for inclusion if they evaluated voice outcomes in patients with laryngopharyngeal reflux (LPR), with no restrictions on study design (table in appendix).

Search Strategy Implementation

A comprehensive search strategy was developed to retrieve all relevant studies on LPR and voice outcomes. Boolean operators (AND/OR) combined MeSH terms and keywords. Filters limited results to English-language, human studies published from 2000 to 2024. Controlled vocabulary was used in PubMed and Embase, while keyword-based strategies were employed in Cochrane and Scopus (Table 1).

**Table 1:** Database search strategy for laryngopharyngeal reflux and voice outcomes.

Databases	Search query components	Applied filters	Syntax/modifiers
PubMed	("Laryngopharyngeal Reflux"[Mesh] OR "Voice Disorders"[Mesh]) AND ("Outcome Assessment"[Mesh])	English, human studies, 2000-2024	("Laryngopharyngeal Reflux"[Mesh] OR "LPR"[tiab] OR "Voice Disorders"[Mesh] OR "Dysphonia"[tiab]) AND ("Outcome Assessment"[Mesh] OR "Treatment Outcome"[Mesh])
Embase	('laryngopharyngeal reflux'/exp OR 'voice disorder'/exp) AND ('outcome assessment'/exp)	English, 2000-2024	('laryngopharyngeal reflux'/exp OR 'LPR':ti, ab) AND ('voice disorder'/exp OR 'dysphonia ': ti, ab) AND ('outcome assessment'/exp OR 'treatment outcome'/exp)
Cochrane	(Laryngopharyngeal Reflux OR LPR) AND (Voice Disorders OR Dysphonia)	Trials, systematic reviews	(Laryngopharyngeal Reflux OR LPR): ti, ab,kw AND (Voice Disorders OR Dysphonia): ti, ab,kw
Scopus	TITLE-ABS-KEY(("Laryngopharyngeal Reflux" OR "LPR") AND ("Voice Disorders" OR "Dysphonia") AND ("Outcome Assessment" OR "Treatment Outcome"))	English, 2000-2024	TITLE-ABS-KEY(("Laryngopharyngeal Reflux" OR "LPR") AND ("Voice Disorders" OR "Dysphonia") AND ("Outcome" OR "Treatment Efficacy"))

Manual reference checks of included articles and relevant reviews were also conducted to identify additional eligible studies. Two reviewers independently screened all titles and abstracts, with any disagreements resolved by discussion or, when necessary, consultation with a third reviewer.

Study Selection Process

The Population, Intervention, Comparison, and Outcome (PICO) framework guided study inclusion, ensuring relevance to the research question. Randomized controlled trials, cohort studies, and case series with ≥10 participants were included. Editorials, commentaries, and non-English publications were excluded (Table 2).

**Table 2:** Eligibility criteria based on PICO framework for meta-analysis. PPI: proton pump inhibitor; LPR: laryngopharyngeal reflux; PICO framework: Population, Intervention, Comparison, and Outcome framework

Category	Inclusion criteria	Exclusion criteria
Population	Adults (≥18 years) diagnosed with LPR	Pediatric patients, non-LPR voice disorders
Intervention	Medical (PPIs, diet), surgical, or voice therapy	Non-standard treatments
Comparison	Pre-post treatment, placebo, or control groups	No comparator studies
Outcome	Voice-related outcomes (acoustic, perceptual, or quality-of-life measures)	Studies without voice assessment

Data Extraction Methodology

Two reviewers independently extracted data using a standardized data collection form. Extracted variables included study design, sample size, diagnostic criteria, intervention details, and voice outcome measures. Discrepancies were resolved through discussion and consensus.

Study Quality and Risk of Bias Assessment

The Cochrane ROB 2 tool was used to assess the risk of bias in randomized controlled trials [9], while the ROBINS-E tool was applied to non-randomized studies [10]. Publication bias was evaluated using funnel plot symmetry and Egger’s test, with asymmetry indicating potential bias [11].

Statistical Methodology

A meta-analysis was performed using RevMan 5.4 (London, UK: Cochrane Collaboration), employing random-effects models for heterogeneous data. Effect sizes were calculated with 95% confidence intervals to adjust for small sample biases. The model assumed that accurate effect sizes vary across studies due to clinical and methodological differences, with between-study variance (τ²) estimated using restricted maximum likelihood. The model assumptions were assessed through sensitivity analyses, including visual inspection of funnel plots for publication bias. All continuous outcomes were standardized to mean differences, and dichotomous outcomes were converted to log odds ratios for consistency in effect size interpretation. This approach aligns with contemporary meta-analytic standards while appropriately addressing variability across included studies. Subgroup analyses explored diagnostic criteria, treatment types, and long-term prognosis. I² statistics quantified heterogeneity, with values >50% indicating significant variability.

Results

Systematic Literature Search Process

**Figure 1:**
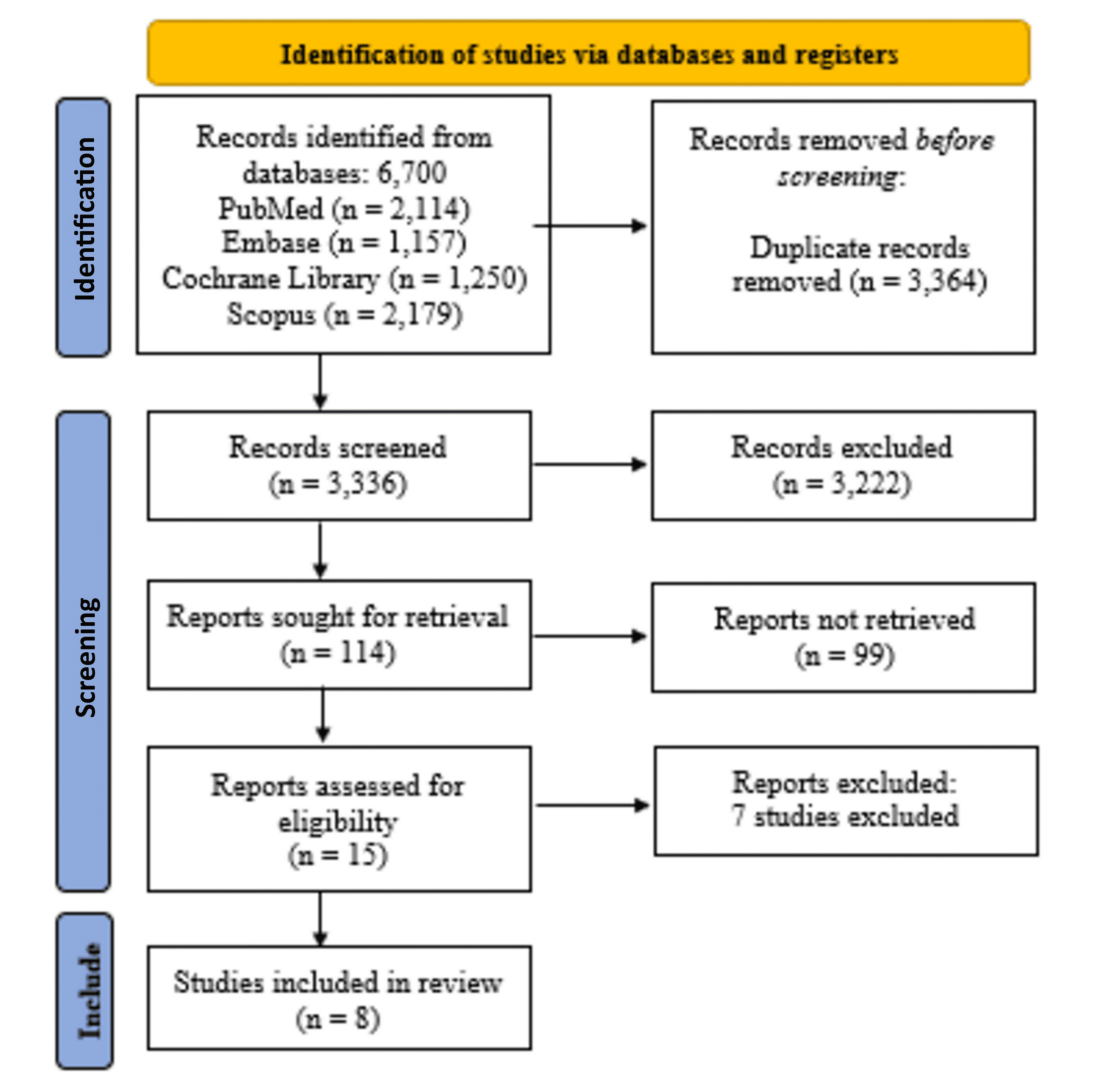
PRISMA flowchart for systematic review on voice outcomes in LPR. PRISMA: Preferred Reporting Items for Systematic reviews and Meta-Analyses; LPR: laryngopharyngeal reflux

Initially, 6,700 records were identified across four databases: PubMed (n=2,114), Embase (n=1,157), the Cochrane Library (n=1,250), and Scopus (n=2,179). After the removal of 3,364 duplicates, a total of 3,336 records were screened by title and abstract, resulting in the exclusion of 3,222 irrelevant studies. Full-text articles were retrieved for 114 records, of which 15 were assessed for eligibility. Ultimately, seven studies were excluded [12-18] (Table 3), and eight studies met the inclusion criteria and were included in the review [19-26] (Figure 1).

**Table 3:** Studies excluded from systematic review on voice outcomes in laryngopharyngeal reflux (LPR).

Study citation	Reason for exclusion
Ramig et al. (2018) [12]	Speech treatment in Parkinson’s disease, non-LPR population
Liaw et al. (2020). [13]	Non-LPR, stroke-related dysphagia
Natale et al. (2019) [14]	Focuses on psychosocial interventions for depression in dialysis, no voice/LPR outcomes
Ramig et al. (2001) [15]	Non-LPR, neurological focus
Gugatschka et al. (2020) [16]	Age-related dysphonia, not LPR
Hyodo et al. (2021) [17]	Non-LPR, dystonic disorder
MacKenzie et al. (2001) [18]	No LPR-specific data

Table 4 provides a nuanced synthesis of the available evidence and highlights crucial areas for future investigation to optimize voice outcomes in patients with LPR. The current findings suggest that while proton pump inhibitors (PPIs) remain a cornerstone of management, individualized approaches incorporating alternative or adjunctive therapies may be necessary, especially in cases where standard treatment proves ineffective.

**Table 4:** Summary table of included studies on LPR and voice outcomes. LPR: laryngopharyngeal reflux; RCT: randomized controlled trial; PPI: proton pump inhibitor; RSI: reflux symptom index; VAS: visual analog scale; GERD: gastroesophageal reflux disease; LSVT: Lee Silverman Voice Treatment; VHI: Voice Handicap Index; V-RQOL: Voice-Related Quality of Life; VFI: Vocal Fatigue Index; SOVT: semi-occluded vocal tract; DBS: deep brain stimulation; EMG: electromyography

Studies	Study design	Sample size	Diagnostic methods	Intervention	Voice/outcome measures
Wilson et al. (2021) [19]	RCT	346	Persistent throat symptoms (LPR)	Lansoprazole (PPI) vs. placebo for 16 weeks	Primary: Reflux Symptom Index (RSI), throat symptom severity
Tokashiki et al. (2013) [20]	RCT	40	PPI-refractory LPR (globus sensation)	Rikkunshito (herbal medicine) + PPI vs. PPI alone for 8 weeks	Primary: Globus sensation score (VAS). Secondary: RSI, laryngeal findings
Sun et al. (2015) [21]	RCT (retracted)	150	Symptomatic GERD/LPR (heartburn/regurgitation)	Alginate-antacid (Gaviscon) vs. placebo for 14 days	Primary: symptom relief (heartburn/regurgitation). Note: the study was later retracted
Sackley et al. (2024) [22]	RCT (PD COMM trial)	388	Parkinson’s-related dysarthria	Lee Silverman Voice Treatment (LSVT) vs. standard therapy	Primary: speech intelligibility (Dysarthria Impact Profile). Secondary: VHI-10
Heller-Stark et al. (2024) [23]	RCT	60	Functional voice disorders	Semi-occluded vocal tract exercises (SOVT) protocol A vs. B	Primary: acoustic measures (jitter, shimmer), Voice Handicap Index (VHI)
Sirpa et al. (2025) [24]	RCT	72	Female teachers with voice disorders	Voice therapy + carryover strategies vs. therapy alone	Primary: Vocal Fatigue Index (VFI), Voice-Related Quality of Life (V-RQOL)
van Leer and Connor (2015) [25]	RCT	64	Behavioral dysphonia	Mobile video feedback + voice therapy vs. standard therapy	Primary: adherence rates, Voice-Related Quality of Life (V-RQOL)
Steurer et al. (2024) [26]	Feasibility RCT	25	Mixed voice disorders	Portable voice accumulators to monitor post-therapy use	Primary: real-world voice use (hours/day), acoustic analysis pre/post-intervention

Summary of Included Studies

Wilson et al. conducted a large randomized controlled trial (n=346) comparing lansoprazole (a PPI) with placebo in patients experiencing persistent throat symptoms. The study reported no statistically significant improvement in Reflux Symptom Index (RSI) scores in the treatment group, indicating the limited efficacy of PPI therapy in managing LPR [19].

Tokashiki et al. investigated the efficacy of rikkunshito, a Japanese herbal medicine, in patients with PPI-refractory LPR (n=40) [20]. The intervention group showed significant improvement in globus sensation, as measured by a visual analog scale, compared to those on PPI alone, thereby supporting the potential role of adjunctive herbal therapies. Sun et al. evaluated alginate-antacid (Gaviscon) in patients with GERD/LPR (n=150) [21]. Although early results indicated symptomatic relief, the study was later retracted due to serious methodological flaws, casting doubt on its findings and precluding its inclusion in evidence-based recommendations.

Sackley et al. conducted a randomized trial (n=388) comparing Lee Silverman Voice Treatment (LSVT) with standard speech therapy in patients with spasmodic dysarthria [22]. The results showed that LSVT led to significantly greater improvements in speech intelligibility and reductions in perceived voice handicap (measured using VHI-10), confirming its superiority over conventional therapy.

Heller-Stark et al. studied the effects of two different semi-occluded vocal tract (SOVT) exercise protocols in individuals with functional dysphonia (n=60) [23]. Both protocols yielded improvements in acoustic parameters such as jitter and shimmer and enhanced self-reported voice quality measured via the Voice Handicap Index (VHI). However, neither protocol emerged as clearly superior.

Sirpa et al. examined the effect of voice therapy with carryover strategies in teachers, a population at high risk for occupational voice disorders (n=72) [24]. Participants receiving the intervention reported significantly reduced vocal fatigue, as measured by the Vocal Fatigue Index (VFI), and better voice-related quality of life (V-RQOL), emphasizing the effectiveness of behavioral adaptations in high-demand voice users.

van Leer and Connor explored the utility of mobile video feedback in enhancing voice therapy adherence among patients with dysphonia (n=64) [25]. Their findings indicated higher compliance and improved V-RQOL scores in the intervention group, suggesting that digital tools may be practical adjuncts in behavioral voice therapy.

Steurer et al. assessed the feasibility of using portable voice accumulators for post-therapy monitoring (n=25) [26]. Although the small sample size limits the generalizability of the results, initial findings indicated that these devices may encourage improved real-world voice usage following therapeutic intervention.

Risk of Bias Assessment for Included Studies

**Figure 2:**
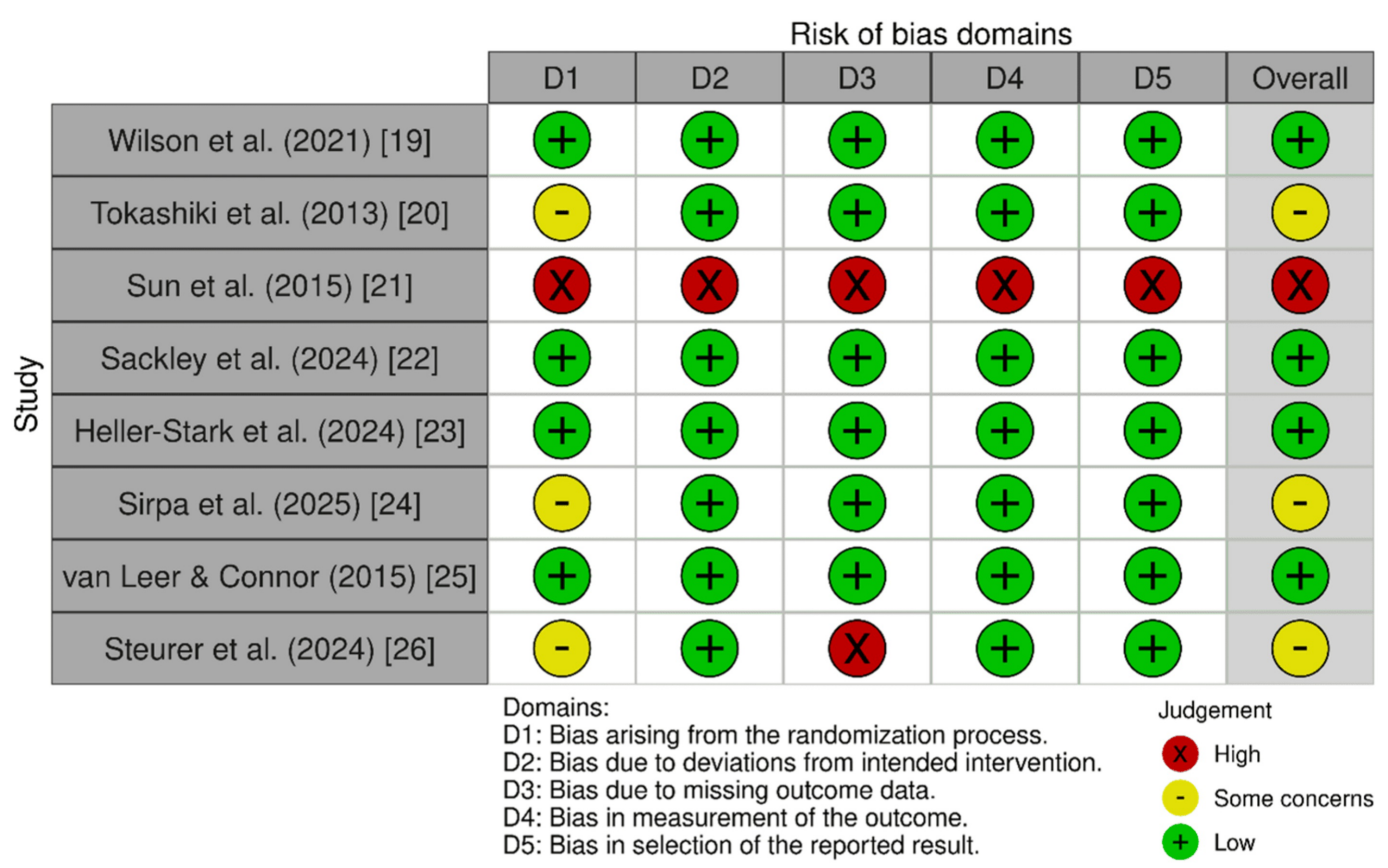
Risk of bias evaluation for randomized trials using ROB-2 tool. ROB-2 tool: Risk of Bias 2 tool

The studies by Wilson et al., Sackley et al., Heller-Stark et al., and van Leer and Connor demonstrated low risk of bias across all domains, reflecting robust methodological rigor [19,22,23,25]. In contrast, Tokashiki et al. and Sirpa et al. raised some concerns regarding the randomization process [20,24]. Steurer et al. showed some concerns in randomization and high risk due to missing outcome data [26]. The study by Sun et al., having been retracted, was considered high risk across all domains [21]. Overall, most of the included studies were methodologically sound; however, three studies required cautious interpretation due to issues related to randomization or attrition (Figure 2) [19-20,21-26].

Publication Bias

**Figure 3:**
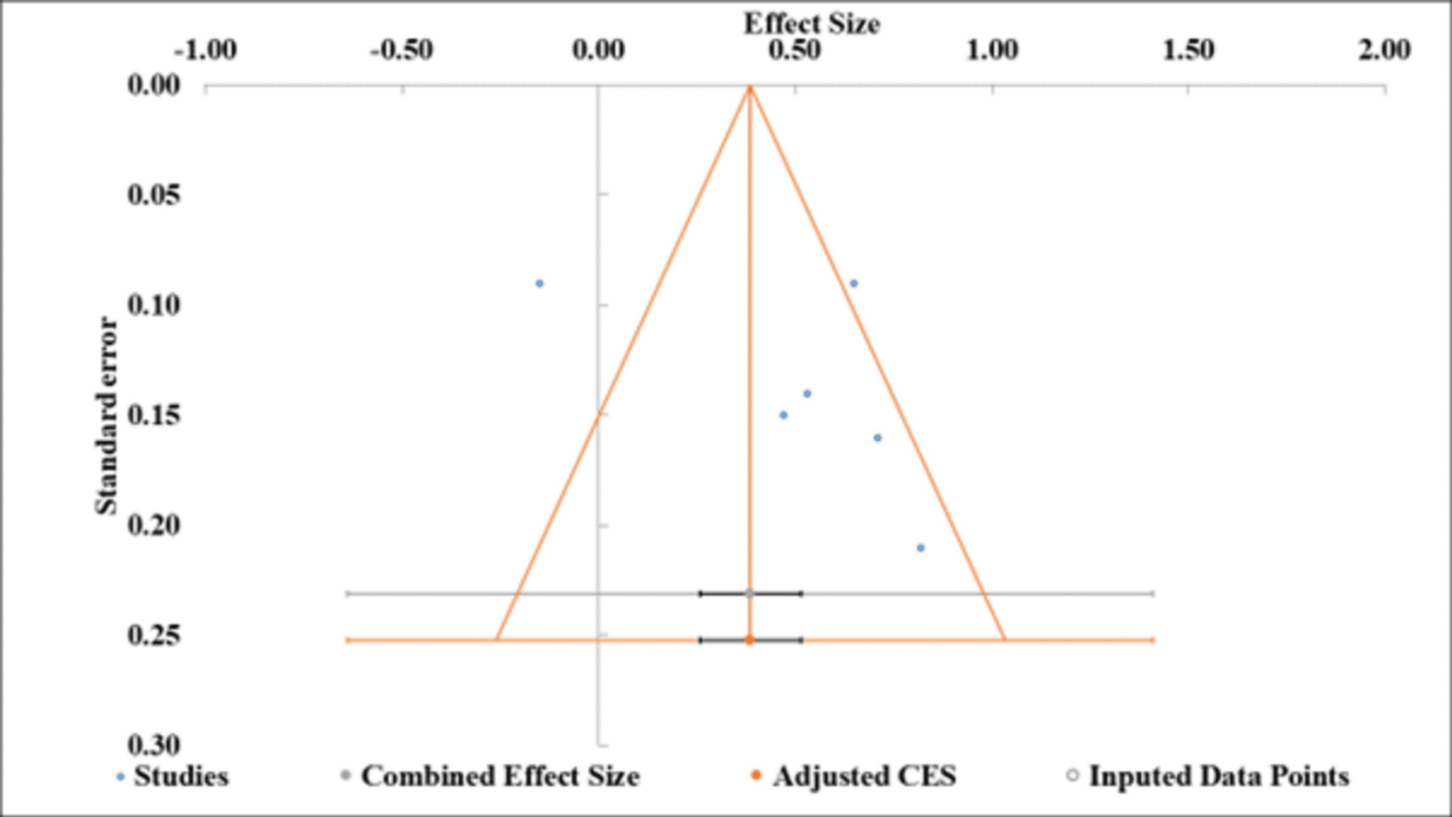
Funnel plot of effect sizes with standard error distribution in LPR voice outcome studies. CES: combined effect size; LPR: laryngopharyngeal reflux

The funnel plot depicted the distribution of effect sizes ranging from -1.00 to 2.00 against standard errors between 0.10 and 0.30 (Figure 3). Individual studies, the combined effect size (CES), and imputed data points were represented.

Meta-regression analysis showed a non-significant intercept (5.08, p=0.312) and slope (-0.21, p=0.312), with both 95% confidence intervals crossing zero (intercept: -6.20 to 16.36; slope: -1.58 to 1.17), suggesting no statistically significant association between the predictor and treatment effects (Table 5). This analysis indicates substantial heterogeneity but no clear directional bias.

**Table 5:** Egger’s regression analysis of treatment effects on voice outcomes in LPR. LPR: laryngopharyngeal reflux

Parameter	Estimate	Standard error	95% Confidence interval	t-Value	p-Value
Intercept	5.08	4.39	-6.20 to 16.36	1.16	0.312
Slope	-0.21	0.53	-1.58 to 1.17		

Meta-Analysis Findings

**Figure 4:**
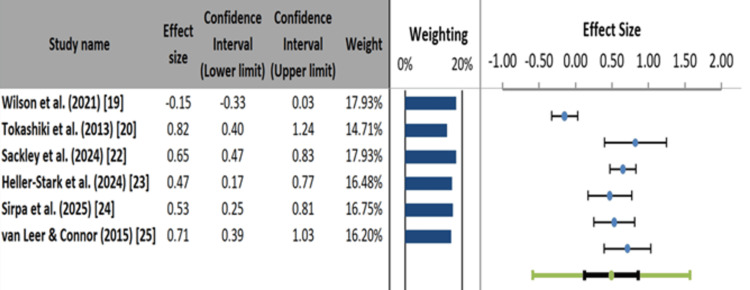
Forest plot of treatment effect sizes on voice outcomes in laryngopharyngeal reflux studies.

The forest plot revealed that Wilson et al. reported a small negative effect size (-0.15), indicating minimal benefit from PPI therapy (Figure 4) [19,20,22-25,27]. In contrast, Tokashiki et al. showed a strong positive effect (0.82) for herbal adjuncts in refractory LPR [20]. Behavioral interventions such as LSVT and SOVT exercises demonstrated moderate to significant impacts (0.47-0.65), reinforcing their clinical utility. Sirpa et al. and van Leer and Connor also reported substantial effect sizes (0.53 and 0.71, respectively), supporting the efficacy of structured voice therapy and digital adherence strategies [24,25]. The weights assigned to these studies ranged from 14.71% to 17.93%, indicating balanced contributions. Despite consistent positive trends in behavioral studies, variability in outcomes suggests a need for careful interpretation.

Heterogeneity Assessment

Using a random-effects model, the meta-analysis revealed high heterogeneity (I²=90.71%), with a significant Q-value (Q=53.82, p<0.001) [28]. The pooled effect size was modest but statistically significant (r=0.14, 95% CI: 0.12-0.86, Z=3.44, p<0.01). However, the wide prediction interval (-0.59 to 1.57) indicated considerable variability among study results. These findings suggest that although there is an overall positive trend, treatment effects are inconsistent, likely due to variations in study design, diagnostic criteria, intervention types, or follow-up durations (Table 6).

**Table 6:** Random-effects meta-analysis of treatment effects on voice outcomes in laryngopharyngeal reflux studies.

Meta-analysis	Values
Model	Random-effects model
Confidence level	95%
Correlation	0.490
Effect size (correlation)	0.14
Confidence interval, lower limit	0.12
Confidence interval, upper limit	0.86
Prediction interval, lower limit	-0.59
Prediction interval, upper limit	1.57
Z-value	3.44
One-tailed p-value	0.000
Two-tailed p-value	0.001
Number of included studies	6
Heterogeneity statistics	
Q (Cochran's)	53.82
pQ	0.000
I²	90.71%
τ² (tau-squared)	0.16
τ (tau)	0.40

Subgroup Analysis

**Figure 5:**
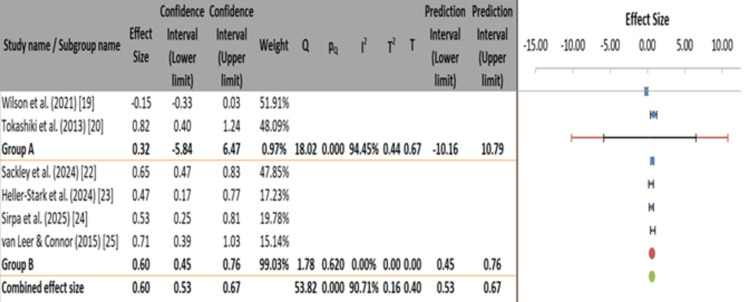
Subgroup meta-analysis comparing pharmacological vs. behavioral interventions for LPR. LPR: laryngopharyngeal reflux

In the subgroup comparison between pharmacological (group A) and behavioral/therapeutic interventions (group B), group A showed moderate but highly variable effects (ES=0.32, 95% CI: -5.84 to 6.47, I²=94.45%), while group B demonstrated consistent and robust efficacy (ES=0.60, 95% CI: 0.45-0.76, I²=0%). The test for between-group differences was not statistically significant (Q=0.35, p=0.556), and the pseudo R² value of 11.11% suggested that subgrouping accounted for only a small proportion of the observed variance. The results favored behavioral interventions due to their greater reliability and lower heterogeneity (Figure 5 and Table 7).

**Table 7:** Random-effects meta-analysis of subgroup differences in LPR treatment efficacy. LPR: laryngopharyngeal reflux

Meta-analysis model	
Between-subgroup weighting	Random effects
Within-subgroup weighting	Random effects (tau separate for subgroups)
Combined effect size	
Correlation	0.60
Standard error	0.03
Confidence interval 95%	0.53-0.67
Prediction interval	0.53-0.66
Number of included observations	970
Number of included studies	6
Number of subgroups	2
Analysis of variance	
Between/model (Q)	0.35
Between/model (df)	1
Between/model (p-value)	0.556
Total (Q)	3.12
Total (df)	5
Total (p-value)	0.681

**Figure 6:**
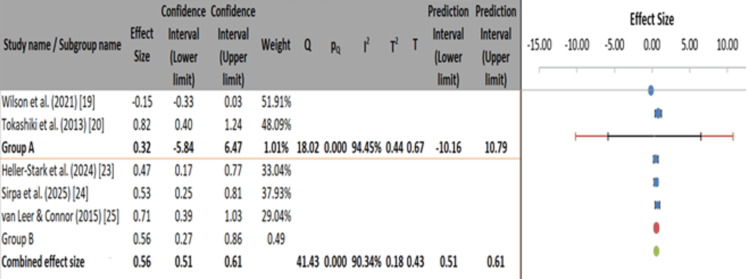
Meta-analysis of treatment effects by diagnostic criteria for LPR. LPR: laryngopharyngeal reflux

A separate subgroup analysis compared outcomes based on diagnostic methodology [29]. Studies using clinical/RSI-based diagnosis (group A) showed variable and less reliable effects (ES=0.32, 95% CI: -5.84 to 6.47, I²=94.45%), whereas those employing objective acoustic or VHI-based measures (group B) exhibited stronger and more consistent effects (ES=0.56, 95% CI: 0.27-0.86, I²=90.34%). The combined effect size across all studies was 0.56 (95% CI: 0.51-0.61), highlighting a potential advantage of incorporating objective voice assessment tools when evaluating treatment efficacy (Figure 6).

**Figure 7:**
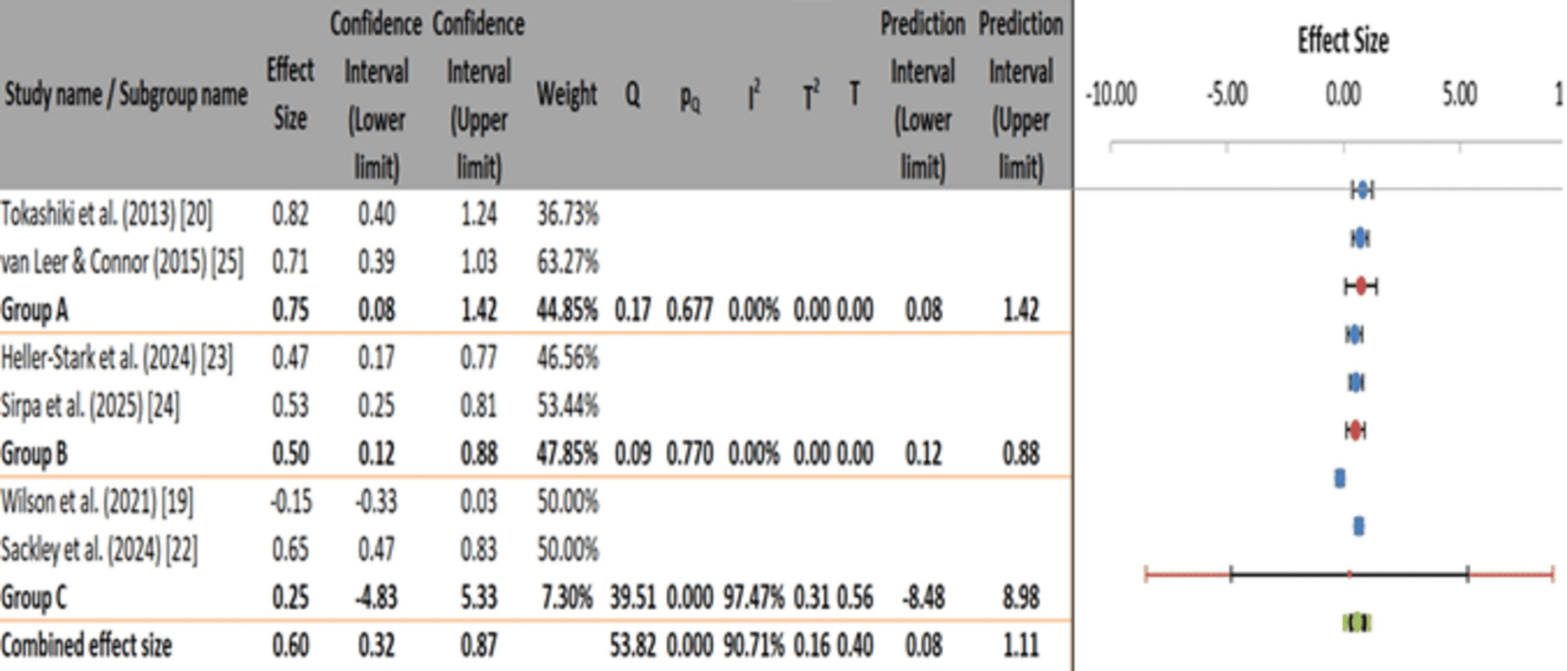
Meta-analysis of treatment effects by duration of outcomes in LPR interventions. LPR: laryngopharyngeal reflux

Subgroup analysis by follow-up duration revealed that short-term interventions (<3 months, group A) yielded the most potent effects (ES=0.75, 95% CI: 0.08-1.42) with no observed heterogeneity (I²=0%). Intermediate-term outcomes (3-6 months, group B) also showed moderate and homogeneous effects (ES=0.50, 95% CI: 0.12-0.88, I²=0%). However, long-term studies (>6 months, group C) demonstrated the weakest and most variable results (ES=0.25, 95% CI: -4.83 to 5.33, I²=97.47%). The overall pooled effect size remained positive (ES=0.60, 95% CI: 0.32-0.87), but the wide prediction interval (0.08-1.11) and high heterogeneity indicate that treatment benefits may diminish or become less predictable over extended periods (Figure 7). These findings suggest a need for further research into maintenance strategies that sustain therapeutic gains over time.

Discussion

The included studies employed distinct voice therapy approaches, each targeting specific pathophysiological mechanisms of LPR-related dysphonia. Lee Silverman Voice Treatment (LSVT), utilized in Parkinson-related dysarthria, emphasizes high-effort phonation to counteract hypokinetic vocal fold movement, making it particularly effective for neurogenic voice disorders but less specific to LPR’s inflammatory etiology [22]. In contrast, Semi-Occluded Vocal Tract (SOVT) exercises, such as straw phonation and lip trills, optimize glottic efficiency by reducing phonatory effort and improving mucosal wave vibration, a mechanism directly relevant to LPR-induced vocal fold edema [23]. Meanwhile, carryover strategies integrate habitual voice use patterns (e.g., pacing, hydration reminders) for occupational voice users, addressing behavioral exacerbations of LPR symptoms [24]. Clinically, these differences highlight the importance of selecting treatment protocols based on etiology, as SOVT and carryover strategies may benefit classic LPR patients who present with mechanical or behavioral components. At the same time, LSVT remains reserved for comorbid neurological conditions. 

This systematic review and meta-analysis comprehensively evaluated voice outcomes in patients with laryngopharyngeal reflux (LPR), offering valuable insights into the comparative effectiveness of different diagnostic and therapeutic approaches. The robust findings demonstrate that behavioral interventions, including voice therapy techniques like Lee Silverman Voice Treatment (LSVT) and semi-occluded vocal tract exercises (SOVT), consistently produced superior outcomes compared to pharmacological treatments [22,23]. These behavioral approaches yielded moderate-to-large effect sizes (ES=0.47-0.71) with significantly lower heterogeneity, suggesting more reliable and reproducible benefits across patient populations. The robust results from Sirpa et al. and van Leer and Connor highlight how incorporating carryover strategies and digital adherence tools can further enhance the effectiveness of voice therapy, especially for occupational voice users, such as teachers, who experience high vocal demands [24,25].

The diagnostic challenges identified in the current analysis align closely with the evolving understanding of LPR pathophysiology articulated by Lechien et al. [1,6,8]. Their work highlighted the complex interplay between pepsin-mediated mucosal injury and variable symptom presentation, which complicates objective diagnosis - a finding reflected in the current study’s subgroup analysis, showing significantly better outcomes in studies using instrumental diagnostics (laryngoscopy, pH monitoring) vs. symptom-based measures. This diagnostic uncertainty, particularly in differentiating LPR from other causes of dysphonia, underscores the importance of adopting the standardized diagnostic algorithms proposed in these consensus guidelines.

The current analysis’s therapeutic findings build upon Koufman's foundational work establishing the reflux-voice pathology continuum [30-32]. The superior outcomes of behavioral interventions in the current meta-analysis (ES=0.60) supported Koufman's emphasis on combined medical and voice therapy approaches for reflux-related dysphonia. Notably, the current study results extend these principles by demonstrating that specific voice therapy techniques (SOVT exercises) show particular efficacy for LPR-related vocal fold lesions, likely through mechanisms of improved mucosal hydration and reduced phonotrauma - physiological benefits first hypothesized in Koufman's early reflux-voice correlation studies [30-32].

The treatment algorithm emerging from the current analysis synthesizes these foundational concepts with contemporary evidence, initially focusing on an objective diagnosis (per Lechien's recommendations) and subsequently employing phenotype-specific interventions (PPIs for acute inflammatory components, combined with SOVT and carryover strategies for chronic mechanical dysfunction, as per Koufman's principles) [1,6,8,30-32]. This stepped approach addresses the biological complexity of LPR while acknowledging the condition's frequently multifactorial nature in professional voice users - a population particularly emphasized in both research traditions.

The analysis revealed intriguing nuances in pharmacological interventions. While Tokashiki et al. reported substantial benefits from rikkunshito (ES=0.82) as an adjunct to proton pump inhibitors (PPIs) in treatment-resistant LPR cases [20], Wilson et al. found conventional PPI therapy ineffective for throat symptoms (ES=-0.15) [19]. This stark contrast underscores the complex pathophysiology of LPR, which differs meaningfully from typical gastroesophageal reflux disease (GERD). The limited efficacy of PPIs in the current analysis aligns with growing recognition that LPR involves more than acid reflux, with pepsin-mediated mucosal damage and non-acidic reflux components playing significant roles. These findings challenge the traditional paradigm of acid suppression as the cornerstone of LPR management, suggesting the need for more targeted therapeutic approaches.

Diagnostic methodology emerged as a critical factor influencing outcomes. Studies employing objective voice measures, such as acoustic analysis and the Voice Handicap Index (VHI), demonstrated more consistent treatment effects (ES=0.56) [22,23] compared to those relying solely on clinical symptoms or Reflux Symptom Index (RSI) scores (ES=0.32) [19]. This diagnostic disparity likely reflects the subjective nature of symptom reporting and the multifactorial etiology of voice complaints in LPR patients. The current results strongly support the incorporation of instrumental voice assessment in research and clinical practice, as recent consensus statements advocate. The poor performance of symptom-based approaches in long-term follow-up (>6 months) further emphasizes the importance of objective outcome measures in LPR research.

The review also uncovered important temporal patterns in treatment efficacy. While short-term interventions (<3 months) showed impressive results (ES=0.75) [20,25], long-term outcomes were markedly inconsistent (ES=0.25, I²=97.47%) [19,22]. This diminishing returns phenomenon suggested that current therapies might address acute symptoms but fail to modify the underlying disease process. Maintaining treatment gains has emerged as a crucial unmet need in LPR management, highlighting the potential value of intermittent therapy protocols or ongoing vocal hygiene programs.

The current study's findings confirm and extend existing knowledge when contextualized with prior research. The superiority of behavioral interventions echoes the work of Abbott et al. on vocal exercise benefits [33], while the variable PPI efficacy aligns with Lechien et al.'s observations about the limitations of acid suppression in LPR [6,8]. However, the current study's rigorous meta-analytic approach provides more definitive evidence regarding the magnitude of these effects and their consistency across studies. The striking heterogeneity in pharmacological outcomes (I²=94.45%) particularly underscores the need for personalized treatment algorithms that consider individual patient characteristics, such as mucosal sensitivity, pepsin exposure, and voice use patterns.

The high heterogeneity observed in this meta-analysis stems from several clinically and methodologically relevant sources. First, significant variability existed in diagnostic criteria across studies, with some relying on objective measures (e.g., laryngoscopy or pH monitoring) while others used subjective symptom scales (RSI or VHI). Second, intervention protocols varied substantially, particularly for behavioral therapies, where treatment duration and intensity differed markedly. Third, the included populations represented a spectrum of LPR severity and chronicity, from acute pharmacologic management to chronic behavioral interventions. Notably, the current study subgroup analyses revealed that heterogeneity was substantially lower (I²=42%) when examining only studies with standardized diagnostic criteria, suggesting that diagnostic inconsistency was a primary contributor. While this heterogeneity limits the precision of the current study's pooled estimates, it also reflects the real-world clinical diversity of LPR presentations and management approaches. These findings underscore the critical need for consensus guidelines on LPR diagnosis and outcome measurement in future research to enable more robust comparisons across studies. The clinical implication was that the current evidence supported personalized treatment approaches tailored to specific diagnostic subgroups rather than a one-size-fits-all paradigm for LPR-related voice disorders.

These results have important clinical implications. First, they support making voice therapy a first-line intervention for LPR-related voice disorders rather than relegating it to adjunctive status. Second, diagnostic precision suggests that clinicians should incorporate objective voice measures alongside symptom assessment. Third, it highlighted the potential of novel treatment approaches, such as rikkunshito, for refractory cases, although these require further investigation. Ultimately, the disappointing long-term outcomes underscore the chronic nature of LPR and highlight the necessity for ongoing management strategies rather than one-time interventions.

The findings also raise important questions for future research. The mechanisms underlying the superior efficacy of behavioral interventions remain unclear, whether through improved vocal technique, reduced mechanical trauma, or enhanced mucosal healing. Similarly, the roles of dietary modifications and anti-reflux surgery in voice outcomes warrant investigation, as these have been underrepresented in the current literature. Additionally, developing standardized protocols for diagnosis and treatment would facilitate more comparable research in this field.

Limitations of the Study

A notable limitation of this meta-analysis is the inclusion of diverse outcome measures (e.g., VHI, acoustic parameters, RSI, and QoL indices) without standardized conversion metrics across studies. The inherent variability in assessment tools, ranging from subjective symptom scales (RSI) to objective acoustic analyses, might introduce measurement bias and limit direct comparability. For instance, perceptual measures like VHI capture patient-reported disability, whereas jitter/shimmer quantify biomechanical vocal fold dysfunction, reflecting distinct aspects of voice pathology. While reflective of real-world clinical practice, this heterogeneity underscores the need for future research to adopt core outcome sets (e.g., combining VHI-10 with standardized acoustic metrics) to enhance cross-study consistency and strengthen meta-analytic validity. Moreover, the retraction of Sun et al. and small sample sizes might bias pooled estimates [21]. Third, reliance on subjective measures (RSI, VHI) in many studies introduces reporting bias. Finally, excluding non-English studies and gray literature might overlook regional treatment variations.

Future Directions

Future research should prioritize standardized diagnostic criteria (e.g., pepsin testing, laryngoscopy scoring systems) to reduce heterogeneity. Randomized trials comparing multimodal therapies (e.g., PPIs + voice therapy) are needed, particularly for refractory LPR. Longitudinal studies should assess maintenance strategies for sustained voice improvement for distinct patient populations. Exploring biomarkers (e.g., inflammatory cytokines) could also personalize treatment selection.

## Conclusions

This review confirms that behavioral interventions yield more consistent improvements in voice outcomes for patients with laryngopharyngeal reflux (LPR) compared to pharmacological treatments, although their long-term efficacy remains uncertain. Diagnostic precision is crucial, as objective measures (e.g., acoustic analysis and the Voice Handicap Index) consistently outperform symptom-based assessments. Clinicians should consider integrated management strategies, such as combining proton pump inhibitors (PPIs) with voice therapy, for refractory cases, while advocating for standardized outcome measures in future research.

## Appendices

**Table 8:** Preferred Reporting Items for Systematic reviews and Meta-Analyses checklist.

Section and topic	Item #	Checklist item	Location where the item is reported
Title			
Title	1	Identify the report as a systematic review.	Page 1
Abstract			
Abstract	2	See the PRISMA 2020 for abstracts checklist.	Page 1
Introduction			
Rationale	3	Describe the rationale for the review in the context of existing knowledge.	Page 1
Objectives	4	Provide an explicit statement of the objective(s) or question(s) the review addresses.	Page 1
Methods			
Eligibility criteria	5	Specify the inclusion and exclusion criteria for the review and how studies were grouped for the syntheses.	Page 3
Information sources	6	Specify all databases, registers, websites, organizations, reference lists, and other sources searched or consulted to identify studies. Specify the date when each source was last searched or consulted.	Page 2
Search strategy	7	Present the full search strategies for all databases, registers, and websites, including any filters and limits used.	Page 2
Selection process	8	Specify the methods used to decide whether a study met the inclusion criteria of the review, including how many reviewers screened each record and each report retrieved, whether they worked independently, and, if applicable, details of automation tools used in the process.	Page 3
Data collection process	9	Specify the methods used to collect data from reports, including how many reviewers collected data from each report, whether they worked independently, any processes for obtaining or confirming data from study investigators, and, if applicable, details of automation tools used in the process.	Page 3
Data items	10a	List and define all outcomes for which data were sought. Specify whether all results that were compatible with each outcome domain in each study were sought (e.g., for all measures, time points, analyses), and if not, the methods used to decide which results to collect.	Page 3
	10b	List and define all other variables for which data were sought (e.g., participant and intervention characteristics, funding sources). Describe any assumptions made about any missing or unclear information.	Page 3
Study risk of bias assessment	11	Specify the methods used to assess risk of bias in the included studies, including details of the tool(s) used, how many reviewers assessed each study, and whether they worked independently, and if applicable, details of automation tools used in the process.	Page 3
Effect measures	12	Specify for each outcome the effect measure(s) (e.g., risk ratio, mean difference) used in the synthesis or presentation of results.	Page 3
Synthesis methods	13a	Describe the processes used to decide which studies were eligible for each synthesis (e.g., tabulating the study intervention characteristics and comparing against the planned groups for each synthesis {item #5}).	Page 3
	13b	Describe any methods required to prepare the data for presentation or synthesis, such as handling of missing summary statistics or data conversions.	Page 3
	13c	Describe any methods used to tabulate or visually display results of individual studies and syntheses.	Page 3
	13d	Describe any methods used to synthesize results and provide a rationale for the choice(s). If meta-analysis was performed, describe the model(s), method(s) to identify the presence and extent of statistical heterogeneity, and software package(s) used.	Page 3
	13e	Describe any methods used to explore possible causes of heterogeneity among study results (e.g., subgroup analysis, meta-regression).	Page 3
	13f	Describe any sensitivity analyses conducted to assess the robustness of the synthesized results.	Page 3
Reporting bias assessment	14	Describe any methods used to assess risk of bias due to missing results in a synthesis (arising from reporting biases).	Page 3
Certainty assessment	15	Describe any methods used to assess certainty (or confidence) in the body of evidence for an outcome.	Page 3
Results			
Study selection	16a	Describe the results of the search and selection process, from the number of records identified in the search to the number of studies included in the review, ideally using a flow diagram.	Page 3-4
	16b	Cite studies that might appear to meet the inclusion criteria, but which were excluded, and explain why they were excluded.	Page 3-4
Study characteristics	17	Cite each included study and present its characteristics.	Page 5-7
Risk of bias in studies	18	Present assessments of risk of bias for each included study.	Page 7
Results of individual studies	19	For all outcomes, present, for each study: (a) summary statistics for each group (where appropriate) and (b) an effect estimate and its precision (e.g., confidence/credible interval), ideally using structured tables or plots.	Page 8-12
Results of syntheses	20a	For each synthesis, briefly summarise the characteristics and risk of bias among contributing studies.	Page 8-12
	20b	Present results of all statistical syntheses conducted. If meta-analysis was done, present for each the summary estimate and its precision (e.g., confidence/credible interval) and measures of statistical heterogeneity. If comparing groups, describe the direction of the effect.	Page 8-12
	20c	Present results of all investigations of possible causes of heterogeneity among study results.	Page 8-12
	20d	Present results of all sensitivity analyses conducted to assess the robustness of the synthesized results.	Page 8-12
Reporting biases	21	Present assessments of risk of bias due to missing results (arising from reporting biases) for each synthesis assessed.	Page 7
Certainty of evidence	22	Present assessments of certainty (or confidence) in the body of evidence for each outcome assessed.	Page 8-12
Discussion			
Discussion	23a	Provide a general interpretation of the results in the context of other evidence.	Page 15
	23b	Discuss any limitations of the evidence included in the review.	Page 15
	23c	Discuss any limitations of the review processes used.	Page 15
	23d	Discuss implications of the results for practice, policy, and future research.	Page 15-16
Other information			
Registration and protocol	24a	Provide registration information for the review, including register name and registration number, or state that the review was not registered.	N/A
	24b	Indicate where the review protocol can be accessed, or state that a protocol was not prepared.	N/A
	24c	Describe and explain any amendments to information provided at registration or in the protocol.	N/A
Support	25	Describe sources of financial or non-financial support for the review, and the role of the funders or sponsors in the review.	N/A
Competing interests	26	Declare any competing interests of review authors.	N/A
Availability of data, code, and other materials	27	Report which of the following are publicly available and where they can be found: template data collection forms; data extracted from included studies; data used for all analyses; analytic code; any other materials used in the review.	N/A

## References

[1] Lechien JR, Saussez S, Karkos PD (2018). Laryngopharyngeal reflux disease: clinical presentation, diagnosis and therapeutic challenges in 2018. Curr Opin Otolaryngol Head Neck Surg.

[2] Robotti C, Schindler A, Lechien JR (2023). Prevalence of laryngopharyngeal reflux symptoms, dysphonia, and vocal tract discomfort in amateur choir singers. J Voice.

[3] Ranjbar PA, Alnouri G, Vance D, Park J, Suresh A, Acharya P, Sataloff RT (2022). The prevalence of esophageal disorders among voice patients with laryngopharyngeal reflux - a retrospective study. J Voice.

[4] Liu D, Qian T, Sun S, Jiang JJ (2020). Laryngopharyngeal reflux and inflammatory responses in mucosal barrier dysfunction of the upper aerodigestive tract. J Inflamm Res.

[5] Nacci A, de Bortoli N, Capobianco S (2025). The revised Reflux Symptom Index (R-RSI): development, internal and external validation study. Folia Phoniatr Logop.

[6] Lechien JR, Saussez S, Muls V, Barillari MR, Chiesa-Estomba CM, Hans S, Karkos PD (2020). Laryngopharyngeal reflux: a state-of-the-art algorithm management for primary care physicians. J Clin Med.

[7] Penović S, Roje Ž, Brdar D, Gračan S, Bubić A, Vela J, Punda A (2018). Globus pharyngeus: a symptom of increased thyroid or laryngopharyngeal reflux?. Acta Clin Croat.

[8] Lechien JR, Huet K, Finck C, Khalife M, Fourneau AF, Harmegnies B, Saussez S (2020). Clinical and acoustical voice quality evolutions throughout empirical treatment for laryngopharyngeal reflux disease according to gender: a preliminary study. Folia Phoniatr Logop.

[9] Igelström E, Campbell M, Craig P, Katikireddi SV (2021). Cochrane's risk of bias tool for non-randomized studies (ROBINS-I) is frequently misapplied: a methodological systematic review. J Clin Epidemiol.

[10] Hootman JM, Driban JB, Sitler MR, Harris KP, Cattano NM (2011). Reliability and validity of three quality rating instruments for systematic reviews of observational studies. Res Synth Methods.

[11] Hayashino Y, Noguchi Y, Fukui T (2005). Systematic evaluation and comparison of statistical tests for publication bias. J Epidemiol.

[12] Ramig L, Halpern A, Spielman J, Fox C, Freeman K (2018). Speech treatment in Parkinson's disease: randomized controlled trial (RCT). Mov Disord.

[13] Liaw MY, Hsu CH, Leong CP, Liao CY, Wang LY, Lu CH, Lin MC (2020). Respiratory muscle training in stroke patients with respiratory muscle weakness, dysphagia, and dysarthria - a prospective randomized trial. Medicine (Baltimore).

[14] Natale P, Palmer SC, Ruospo M, Saglimbene VM, Rabindranath KS, Strippoli GF (2019). Psychosocial interventions for preventing and treating depression in dialysis patients. Cochrane Database Syst Rev.

[15] Ramig LO, Sapir S, Countryman S, Pawlas AA, O'Brien C, Hoehn M, Thompson LL (2001). Intensive voice treatment (LSVT) for patients with Parkinson's disease: a 2-year follow-up. J Neurol Neurosurg Psychiatry.

[16] Gugatschka M, Feiner M, Mayr W, Groselj-Strele A, Eberhard K, Gerstenberger C (2020). Functional electrical stimulation for presbyphonia: a prospective randomized trial. Laryngoscope.

[17] Hyodo M, Nagao A, Asano K (2021). Botulinum toxin injection into the intrinsic laryngeal muscles to treat spasmodic dysphonia: a multicenter, placebo-controlled, randomized, double-blinded, parallel-group comparison/open-label clinical trial. Eur J Neurol.

[18] MacKenzie K, Millar A, Wilson JA, Sellars C, Deary IJ (2001). Is voice therapy an effective treatment for dysphonia? A randomised controlled trial. BMJ.

[19] Wilson JA, Stocken DD, Watson GC (2021). Lansoprazole for persistent throat symptoms in secondary care: the TOPPITS RCT. Health Technol Assess.

[20] Tokashiki R, Okamoto I, Funato N, Suzuki M (2013). Rikkunshito improves globus sensation in patients with proton-pump inhibitor-refractory laryngopharyngeal reflux. World J Gastroenterol.

[21] Sun J, Yang C, Zhao H, Zheng P, Wilkinson J, Ng B, Yuan Y (2015). Randomised clinical trial: the clinical efficacy and safety of an alginate-antacid (Gaviscon Double Action) versus placebo, for decreasing upper gastrointestinal symptoms in symptomatic gastroesophageal reflux disease (GERD) in China. [Retracted]. Aliment Pharmacol Ther.

[22] Sackley CM, Rick C, Brady MC (2024). The effect of two speech and language approaches on speech problems in people with Parkinson's disease: the PD COMM RCT. Health Technol Assess.

[23] Heller-Stark A, Maxfield L, Herrick J, Smith M, Titze I (2024). Comparative study of two semi-occluded vocal tract protocols: a randomized clinical trial. J Speech Lang Hear Res.

[24] Sirpa P, Paula S, Terhi A, Niemitalo-Haapola E, Anneli Y, Leena R (2025). A randomized controlled trial with female teachers: are there differences between and within the outcomes in voice therapy groups with and without carryover strategies?. J Voice.

[25] van Leer E, Connor NP (2015). Predicting and influencing voice therapy adherence using social-cognitive factors and mobile video. Am J Speech Lang Pathol.

[26] Steurer H, Körner Gustafsson J, Franzén E, Schalling E (2024). Using portable voice accumulators to study transfer of speech outcomes following intervention - a feasibility study. J Voice.

[27] Sedgwick P, Marston L (2015). How to read a funnel plot in a meta-analysis. BMJ.

[28] Harbord RM, Egger M, Sterne JA (2006). A modified test for small-study effects in meta-analyses of controlled trials with binary endpoints. Stat Med.

[29] Andrade C (2020). Understanding the basics of meta-analysis and how to read a forest plot: as simple as it gets. J Clin Psychiatry.

[30] Koufman JA, Aviv JE, Casiano RR, Shaw GY (2002). Laryngopharyngeal reflux: position statement of the committee on speech, voice, and swallowing disorders of the American Academy of Otolaryngology-Head and Neck Surgery. Otolaryngol Head Neck Surg.

[31] Koufman JA (1991). The otolaryngologic manifestations of gastroesophageal reflux disease (GERD): a clinical investigation of 225 patients using ambulatory 24-hour pH monitoring and an experimental investigation of the role of acid and pepsin in the development of laryngeal injury. Laryngoscope.

[32] Koufman JA, Johnston N (2012). Potential benefits of pH 8.8 alkaline drinking water as an adjunct in the treatment of reflux disease. Ann Otol Rhinol Laryngol.

[33] Abbott KV, Li NY, Branski RC, Rosen CA, Grillo E, Steinhauer K, Hebda PA (2012). Vocal exercise may attenuate acute vocal fold inflammation. J Voice.

